# Laboratory Testing for Middle East Respiratory Syndrome Coronavirus, California, USA, 2013–2014

**DOI:** 10.3201/eid2109.150476

**Published:** 2015-09

**Authors:** Mahtab Shahkarami, Cynthia Yen, Carol Glaser, Dongxiang Xia, James Watt, Debra A. Wadford

**Affiliations:** California Department of Public Health, Richmond, California, USA

**Keywords:** MERS, MERS-CoV, MERS coronavirus, Middle East respiratory syndrome, novel coronavirus, California, surveillance, laboratory testing, viruses, travel-associated, imported, infection control

## Abstract

Since Middle East respiratory syndrome coronavirus (MERS-CoV) first emerged, the California Department of Public Health has coordinated efforts to identify possible cases in travelers to California, USA, from affected areas. During 2013–2014, the department investigated 54 travelers for MERS-CoV; none tested positive, but 32 (62%) of 52 travelers with suspected MERS-CoV had other respiratory viruses.

Middle East respiratory syndrome coronavirus (MERS-CoV) has been a global concern since its discovery in Saudi Arabia in 2012. As of April 29, 2015, >1,100 confirmed MERS cases and >420 associated deaths had occurred globally; all cases were linked to the Middle East (*1*). Importation of MERS-CoV by travelers from the Arabian Peninsula to regions outside the Middle East has been documented (*2*). In May 2014, the first 2 cases of MERS in the United States were identified in unrelated travelers from Saudi Arabia (*3*).

Each year, an estimated 16 million international travelers visit California (*4*), of whom 225,000 are visitors from the Middle East (*5*); thus, a risk exists for importation of MERS-CoV into California. Furthermore, global events such as the annual Hajj and Umrah pilgrimages draw 11,000 Americans to Saudi Arabia each year (*6*).

Because of the possible risk for disease transmission, the Centers for Disease Control and Prevention (CDC) and the World Health Organization have issued MERS-CoV travel advisories for pilgrims traveling to Saudi Arabia (*7*,*8*). In the fall of 2012, the California Department of Public Health (CDPH) addressed the risk of MERS-CoV importation and convened a working group composed of clinicians, laboratory staff, infection control experts, emergency operations staff, and information officers. This working group regularly reviewed the CDC and World Health Organization updates, scientific publications, and laboratory logistics, and took steps at the state level to prepare for MERS. CDPH developed and disseminated guidance on surveillance, specimen collection for laboratory testing, infection control, and contact tracing (*9*). A CDPH clinician was available around the clock 7 days a week to assist with individual suspected cases of MERS.

## The Study

CDPH created a laboratory testing plan to detect or rule out MERS-CoV infection in patients who, after review by CDPH clinicians, met specific clinical and travel criteria, per CDC case definitions (*10*), to be considered a patient under investigation (PUI). Once a MERS PUI was identified, the patient’s specimens were transported from the hospital or local public health laboratory to CDPH in Richmond, California, for MERS-CoV testing. Specimens tested for each PUI consisted of >1 of the following: upper respiratory tract sample (nasopharyngeal and oropharyngeal swab specimens), lower respiratory tract sample (sputum and lower respiratory tract aspirates or washes), serum, or stool. Time from specimen collection to receipt at CDPH was up to 48 hours for most PUIs (37/52 [71%]). Because subsequent steps in infection control and patient management heavily depended on the test results, MERS-CoV testing at CDPH was expedited; the typical turnaround time was 4 hours from receipt of specimens to reporting of results.

During February–June 2013, specimens from MERS PUIs were tested at CDPH for MERS-CoV by using an in-house real-time reverse transcription PCR (rRT-PCR) assay that amplified the following 3 targets in the MERS-CoV genome: UpE, N2, and N3 (*11*). CDPH implemented CDC’s Novel Coronavirus 2012 Real-Time RT-PCR Assay protocol subsequent to its Emergency Use Authorization by the US Food and Drug Administration in June 2013 (*12*).

For persons with a suspected past MERS-CoV infection, CDPH sent serum specimens to CDC for MERS-CoV serologic testing. Once MERS-CoV infection was ruled out, CDPH tested the remaining respiratory specimens from MERS PUIs for other respiratory pathogens. Specimens were tested by real-time PCR and rRT-PCR for the following agents (*13*): influenza A and B viruses, human metapneumovirus, respiratory syncytial virus, adenovirus, parainfluenza virus (types 1, 2, 3, and 4), enterovirus, rhinovirus, and *Mycoplasma* spp. If an adequate amount of specimen remained, specimens were also tested for the presence of human coronaviruses 229E, OC43, NL63, and HKU1 by rRT-PCR (*13*).

During February 2013–November 2014, CDPH investigated 54 MERS PUIs in California, of whom 52 (total of 188 specimens) had testing conducted by CDPH and 2 had testing conducted by CDC (Figure). The median age for MERS PUIs was 53 years (range 10 months–89 years), and 57% were male and 43% female. A total of 51 (94%) MERS PUIs reported travel from the Middle East, and 2 (4%) were secondary contacts of travelers to the Middle East. A MERS PUI short form or equivalent was submitted to CDC and reported the following clinical outcomes for 42 MERS PUIs: 30 (71%) hospitalized, 11 (26%) admitted to an intensive care unit, 6 (14%) intubated, 21 (50%) received a diagnosis of pneumonia, and 5 (12%) received a diagnosis of acute respiratory distress syndrome.

**Figure:**
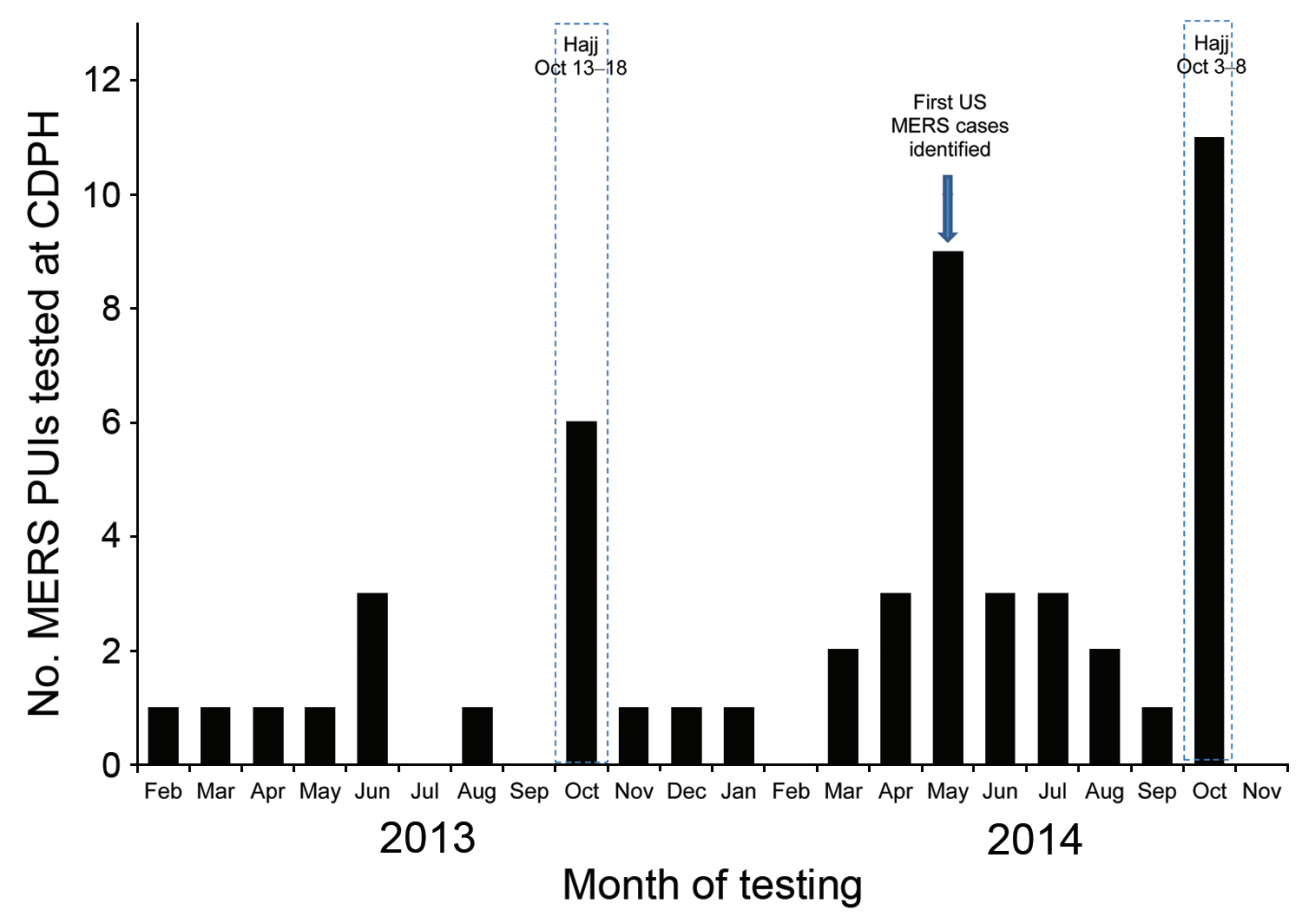
Middle East respiratory syndrome (MERS) coronavirus patients under investigation (PUIs) tested at the California Department of Public Health (CDPH), 2013–2014. Months during which the Hajj takes place are delineated by dashed lines.

One or more respiratory viruses were detected in 32 (62%) of the 52 MERS PUIs tested by CDPH; 5 of the 32 patients had a co-infection with rhinovirus plus another respiratory virus. Influenza, the most commonly identified respiratory agent, was detected in 18 (35%) of the 52 MERS PUIs tested by CDPH (Table). *Mycoplasma* spp. was not detected in any specimen tested.

**Table:** Respiratory viruses detected in MERS patients under investigation tested by California Department of Public Health, 2013–2014*

Virus detected	No. patients, N = 52	% Positive
Influenza only	14	27
Influenza A (H3)	10	19
Influenza A(H1N1)pdm09	3	6
Influenza B	1	2
Noninfluenza only	13	25
Respiratory syncytial virus	1	2
Parainfluenza 3	2	4
Rhinovirus	3	6
Enterovirus	2	4
Human coronavirus 229E	2	4
Adenovirus	3	6
Co-infection	5	10
Influenza A (H3) and rhinovirus	1	2
Influenza A(H1N1)pdm09 and rhinovirus	1	2
Influenza B and rhinovirus	2	4
Parainfluenza 3 and rhinovirus	1	2
No. patients with detected virus	32	62

*MERS, Middle East respiratory syndrome.

The frequency of MERS PUIs tested by CDPH varied with no apparent seasonality, except for the weeks following the Hajj in 2013 and 2014 (Figure). CDPH also noted an increase in reported MERS PUIs in May 2014 (n = 9) after announcement of the first detected MERS cases in the United States (*3*). This increase likely resulted from media reports that heightened the level of concern among the public and health care workers, which increased the number of suspect MERS cases that CDPH and local partners had to evaluate for subsequent MERS-CoV testing.

## Conclusions

As of May 7, 2015, MERS-CoV had not been detected in California. However, MERS-CoV poses a potential threat to global public health because MERS cases continue to be reported in Saudi Arabia, and the reservoir for the virus remains unclear, although camels have been implicated in disease transmission (*14*). CDPH has established a coordinated statewide system working with local partners to identify potential MERS cases in California travelers returning from MERS-affected regions and their contacts. CDPH has investigated and conducted laboratory testing on >50 MERS PUIs and identified a respiratory virus in 62% of those patients, 35% of which were positive for influenza virus. The high rate of influenza detection underscores the need for all travelers to be immunized for influenza. CDPH continues to evaluate each MERS PUI and expedite MERS-CoV laboratory testing so that prompt implementation of containment procedures and contact investigations may proceed if needed.
